# Illustrations of the Elementary Forms of Disease

**Published:** 1834-07-01

**Authors:** 


*Illustrations of the Elementary Forms of Disease.* By ROBERT CARSWELL, M.D., Professor of Pathological Anatomy in the University of London, &c. Fasciculus V. *Softening.*—London, 1834.

THIS fasciculus is not inferior to the preceding ones, and those who have seen any one of them will immediately allow that panegyric can go no higher. Dr. Carswell's investigations are still characterized by the same spirit of impartiality, and his plates continue to have the same correctness of drawing, and the same exquisite beauty of colouring. His observations on the erosions of the stomach after death by the gastric juice, which have so frequently been mistaken by the French pathologists for the effects of disease, are extremely judicious and convincing; but it is not necessary to quote them here, as we gave a summary of his opinions on this point in our last Number, in the review of the Cyclopædia of Medicine.

In speaking of inflammatory softening of the cellular tissue, our author says,

"Muscular tissue is also easily torn or separated into shreds, in consequence of softening of the interstitial cellular tissue by which its fibres are united. This is often observed in the muscles of voluntary motion, and sometimes in the heart. The easy separation of the serous and mucous membranes is always the consequence of inflammatory softening of their connecting cellular tissue, and the degree of facility with which their separation is effected, affords a ready means of estimating the degree and extent of the inflammation to which this tissue had been subjected. Cases of peritonitis and meningitis occur which would escape our *post-mortem* researches but for this state of softening of the cellular tissue. In such cases there may be little or no increase of vascularity; and perhaps only a slight serous effusion, which may be overlooked, or, if observed, can afford no idea of the degree and extent of this morbid condition of the cellular tissue. Both are readily ascertained in peritonitis, by making a circular incision of the peritoneum, and then pulling the intestine in a longitudinal direction. The intestine is thus as it were unsheathed, by the gradual separation of its muscular coat from the peritoneum. In meningitis the blood-vessels are separated from the pia mater in a similar manner."

When these splendid illustrations are completed, they will not merely be indispensable to every medical library, but ought to be found in those magnificent collections of books, where the more opulent patrons of knowledge delight to bring together all the works most conspicuous for their merit, in each department of art, science, and literature.

